# A New Strategy to Reduce Influenza Escape: Detecting Therapeutic Targets Constituted of Invariance Groups

**DOI:** 10.3390/v9030038

**Published:** 2017-03-02

**Authors:** Julie Lao, Anne Vanet

**Affiliations:** 1 Paris Diderot University, University Sorbonne Paris Cité, F-75013 Paris, France; julie.lao@etu.univ-paris-diderot.fr; 2 Epôle de Génoinformatique, Institut Jacques Monod, UMR7592, CNRS, F-75013 Paris, France

**Keywords:** resistance, bioinformatics, influenza, hemagglutinin, drug targets, synthetic lethality

## Abstract

The pathogenicity of the different flu species is a real public health problem worldwide. To combat this scourge, we established a method to detect drug targets, reducing the possibility of escape. Besides being able to attach a drug candidate, these targets should have the main characteristic of being part of an essential viral function. The invariance groups that are sets of residues bearing an essential function can be detected genetically. They consist of invariant and synthetic lethal residues (interdependent residues not varying or slightly varying when together). We analyzed an alignment of more than 10,000 hemagglutinin sequences of influenza to detect six invariance groups, close in space, and on the protein surface. In parallel we identified five potential pockets on the surface of hemagglutinin. By combining these results, three potential binding sites were determined that are composed of invariance groups located respectively in the vestigial esterase domain, in the bottom of the stem and in the fusion area. The latter target is constituted of residues involved in the spring-loaded mechanism, an essential step in the fusion process. We propose a model describing how this potential target could block the reorganization of the hemagglutinin HA2 secondary structure and prevent viral entry into the host cell.

## 1. Introduction

RNA viruses are accountable for multiple outbreaks and severe pandemics. Over 33 million individuals are infected with human immunodeficiency virus (HIV) and over 170 million have hepatitis C. Each year, more than 100 million cases of seasonal flu are recorded. Every century, influenza A strains (the most common influenza in human beings) are answerable for at least one pandemic, the best known and the most deadly of the twentieth century was generated by the 1918 Spanish flu. Even recently, a new strain of influenza A H1N1 triggered another pandemic. Several drugs against influenza viruses have been developed and are presently administered. Adamantanes (amantadine and rimantadine) target the M2 protein that constitutes an ion channel: once blocked, they render the hemagglutinin (HA) protein, one of the two glycoproteins at the surface of the virus, non-functional [1,2]. It is important to note that the first action of the M2 protein is to prevent unpacking of the virus particle. Oseltamivir and zanamivir inhibit neuraminidase (NA) one of the two surface glycoproteins of the virus [2].

Despite the existence of treatments, RNA viruses generally represent a serious public health problem. Indeed, their high mutation rate allows them to rapidly acquire resistances to these treatments [3]. Since 2003, most circulating strains of the seasonal influenza A H3N2 now exhibit resistance to adamantanes [4]. Moreover, the existence of double resistant A H1N1 strains to adamantanes as well as to NA inhibitors restrict the finding of effective treatments [5]. Resistance to these medications could be the consequence of a single amino acid (AA) mutation [3,6,7]. To prevent the emergence of resistance, a strategy targeting invariant AAs allowed the development of new alternative anti-flu treatments [8]. Indeed, mutations in highly conserved positions lead to a deterioration or loss of biological functions and could thereby render the virus unviable [9]. However, invariant positions alone cannot constitute binding sites for a drug because of their very small number. To find the best binding sites, we applied a workflow which consists in also looking for synthetic lethals (SLs) to find durable therapeutic targets accessible to a drug [10,11]. SLs represent mutations that are not lethal but when combined make the virus unviable. These SLs have been scrutinized to search for anticancer drugs [12,13] and anti-HIV agents. Thus, targets constituted of invariant AAs and SLs detected with this workflow should, once mutated by the virus or blocked by a drug, induce the loss of an important biological function of the virus (Figure 5 of [14]).

To test this workflow, we investigated the influenza A H1N1 and H3N2 HA proteins. This homotrimeric protein binds to sialic acid on the surface of its host cells, allowing entry of the virus by endocytosis [15,16,17]. We first aligned 13,793 AA sequences of H1N1 HA and 13,290 AA sequences of H3N2 HA. From these alignments, we determined invariant positions and pairs of SLs using statistical tests. Then, we focused on sets of spatially close “SL + Invariants” accessible to the solvent. Our method yielded three “SL + Invariant” groups that could be satisfactory candidates to form pockets for potential small drug molecules, with one group directly impacting the essential mechanism of virus fusion, and another one preventing this mechanism from appearing. Preliminary drug design experiments allow us to consider the possibility of blocking the fusion process by fixing the tripeptide KFE (lysine–phenylalanine–glutamic acid) on one of the described “SL + Invariant” groups.

## 2. Materials and Methods

**Figure 1:**
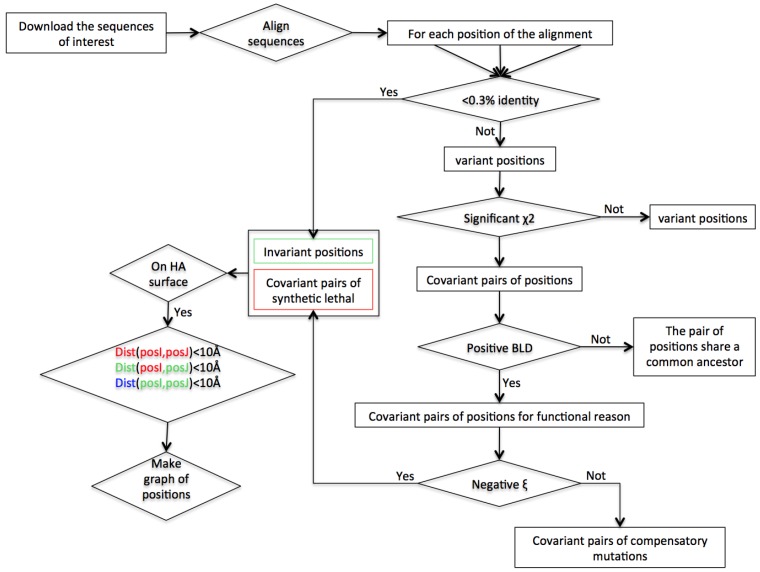
Workflow of the bioinformatic and statistical methods. Data are in rectangles, and processes in diamonds. Dist (posI, posJ) expresses the physical distance between a position I and a position J. The objective of this workflow is to determine the lethal synthetic pairs (in red in the diagram) and the invariant positions (in green in the diagram) nearby to make a graph (Figure 3), where the edge between two invariant positions will be blue, between two positions SL will be red and between an invariant position and a synthetic lethal will be green.

The workflow explaining the different steps composing this method is depicted in Figure 1.

### 2.1. Primary Sequence and Quaternary Structure References

Influenza strains are characterized by a combination of 18 HAs (H1 to H18) and 11 NAs (N1 to N11). To find a primary sequence reference for the influenza HA of strain A H1N1, it is essential to analyze phylogenetic trees of H1 and N1. Indeed the reference sequence to be selected must be representative of all the sequences studied, in other words be one of their common ancestors. HA and NA genes of strain A (H1N1) pdm09 (responsible for the 2009 A H1N1 influenza pandemic) are derived from the common flu virus in pigs and humans. Thus, we selected as reference the primary sequence of the HA of the A/Weiss/43 (H1N1) strain because examination of phylogenetic trees of the HA and NA sequences shows that the Weiss43 sequence is at the junction between sequences of strains infecting humans and pigs [18,19].

**Figure 2:**
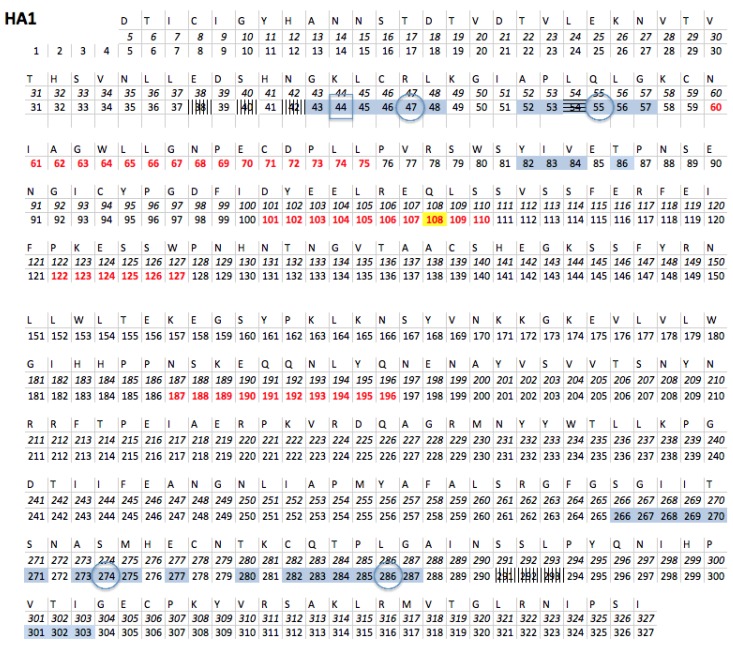

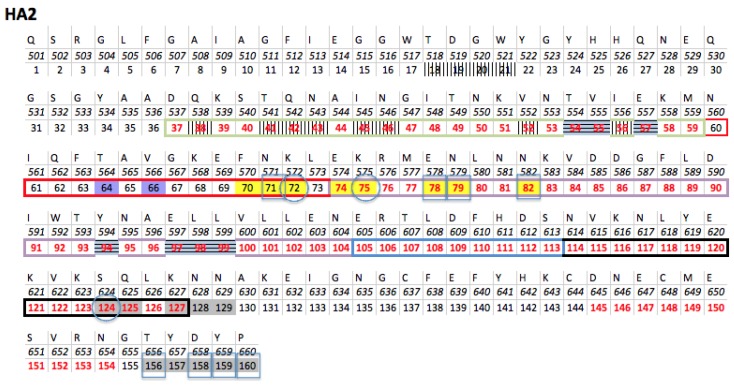
Hemagglutinin (HA) amino acid sequence, helix, loop and target positions. The HA1 and HA2 amino acid sequences of the 3D reference strain 1RU7.pdb are presented in this figure. The numbering of the positions used here is in italics and corresponds to that used in the protein databank (PDB) file of the three-dimensional reference sequence (1RU7.pdb). The second numbering links the PDB numbering with the ones of the H1N1 HA1 and HA2 positions used in the literature. The positions in red belong to a helical secondary structure. HA2 contains several helices: zone A is boxed in light green, loop B is boxed in red, zone C is boxed in violet, Kink is blue boxed, and zone D is boxed in black. The H1N1 targets: the third target (#83) described in this study is highlighted in yellow, the first one (#9) in light blue and the second one (#67) in grey. The target linked by the *tert*-butylhydroquinone (TBHQ) molecule in [24] is horizontally hatched. The target linked by the monoclonal antibody (Mab) C179 in [25] is vertically hatched. The invariant positions located in our target are surrounded by a square and the SL positions by a circle.

Concerning H3N2, the J02090 sequence named Aichi2 from Japan [20] was chosen as primary reference sequence. Indeed it is proposed as the first H3N2 flu virus to infect human [21]. The 3HMG quaternary structure [22] from the protein databank (PDB) was chosen as tridimensional reference sequence for H3N2 and corresponds to the best resolution of the tri-dimensional structure of the Aichi2 strain. Analysis of the H1N1 HA 3D structures downloaded from the PDB indicates that 1RU7 [23] is the one that has the nearest primary sequence to our sequence reference. Biological tests (antibody recognition) have proven that the protein used to determine the quaternary structure is indeed a HA [23]. Figure 2 shows the amino acid sequence of HA H1N1 1RU7.

### 2.2. Construction of Sequence Dataset

From the website “Influenza Research Database” (fludb.org), 13,793 nucleotide sequences of genes encoding HA of patients infected with influenza A H1N1, were downloaded on 15 March 2016. The following parameters were used: pH1N1 (p for pandemic) excluding laboratory strains. From the same website, 13,290 nucleotide sequences of genes encoding HA from H3N2 strains were downloaded on 20 December 2016.

Sequences are aligned at the 5’-end: they all have variable lengths due to sequencing deletion errors of some of them. Rather than using multiple alignment methods that are extremely time-consuming due to the necessity to align over ten thousand sequences, we defined two filters to clean up our dataset of its background noise. As stated above, the primary sequence of the H1N1 Weiss43 HA was adopted as reference sequence and Aichi2 for H3N2. These sequences are 1701 nucleotides long. The first filter excludes sequences of another length than that of the reference sequence. This filter sets the new sequence dataset at 11,769 HA sequences from H1N1 strains and 13,267 for HA sequences from H3N2 strains. The second filter applied eliminates sequences that have a higher number of mutations than three standard deviations of the average number of mutations per sequence.

Once the second filter has been applied, the alignment of HAs of 10,781 H1N1 and 12,225 H3N2 remain. The new set of sequences is then modified to correspond to the residue numbering of the quaternary structure of the reference sequence. Concerning the H1N1 HA alignment, first the signal peptide was not part of the mature protein, and then nucleotides corresponding to its sequence were removed. Second, crystallographers who built the 1RU7 (from PDB) 3D structure, were unable to locate all the residues of the primary sequence of HA. Altogether, four parts of the alignment were deleted and replaced by gaps, from nucleotide 1 to 51, from nucleotide 439 to 441, from nucleotide 1024 to 1032, and finally from nucleotide 1513 to 1701, resulting to a coding sequence of 323 AAs for HA1 and 160 AAs for HA2. Once the peptide signal is cleaved, the coding sequence of the wild type HA1 is 327 AAs and 222 AAs for HA2. It is noteworthy that 4 AAs at the N-terminal end of HA1 are missing as well as 62 AAs at the C-terminal end of HA2. Indeed, this C-terminal end being buried into the cellular/virion membrane fusion, cannot be part of the crystal structure. Concerning H3N2, three parts of the alignment were deleted and replaced by gaps from nucleotide 1 to 47, from nucleotide 1032 to 1034, and finally from nucleotide 1560 to 1701 resulting to a coding sequence of 328 AAs for H3N2 HA1 and 175 for H3N2 HA2.

### 2.3. Identification of Accessible Variant Positions

Only varying residues on the protein surface were taken into consideration. The alignments contain sequencing errors, whose percentage must be defined. In a previous article we defined this number by calculating the mutation rate of the positions necessary for the proper functioning of an active site (HIV protease [10]). This percentage was 0.3% and corresponded to that described in the literature. Then the variable positions are those that are mutated in more than 0.3% of the sequences aligned. To define the accessibility of residues to a ligand that could be a potential future drug, we calculated the surface area accessible to solvents with the ASA software [26] (available on the Ressource Parisienne en Bioinformatique Structurale (RPBS) website [27]) using the 3D 1RU7 structure for H1N1 and 3HMG for H3N2. All residues whose threshold accessibility was superior to 25% were considered accessible.

### 2.4. Identification of Interdependent Positions: χ^2^ Test

The covariation of residues at positions *i* (pos_i_) and *j* (pos_j_) was studied taking into account the nature of the AAs at these positions. From the group of variant positions, we performed a *χ*^2^ test of independence for all pairs of pos_i_, pos_j_ taking into account all possible AA combinations at these positions:

$$\chi^{2}\left(i,j\right)=\underset{n}{\sum }\frac{{\left(n_{obs}-n_{exp}\right)}^{2}}{n_{exp}}$$

*n* = number of pairs of different AAs at positions *i* and *j*.

First, 2000 sets of residues at pos_i_ and pos_j_ were randomly generated to calculate 2000 $\chi_{random}^{2}\left(i,j\right)$. These randomized sets preserved the percentage of each existing AA in the position studied. These 2000 values were then organized in ascending order and partitioned into 20 subsets equal in numbers (5% quantiles). If the $\chi^{2}\left(i,j\right)$ was within the 20th quantile (belongs to the 5% best values), we defined a *p*-value whose value was the $\chi_{random}^{2}\left(i,j\right)$ nearest to $\chi^{2}\left(i,j\right)$.

False positives due to multiple tests were then corrected by readjusting the *p*-values using the false discovery rate (FDR) method (control of the proportion of false positives among significant tests). Positions were considered dependent if the *p*-value obtained was below 0.05. This method was adapted from the Noivirt study [28].

### 2.5. Identification of the Background Linkage Disequilibrium (BLD)

Using sets of DNA sequences, it was possible to determine mutated codons causing synonymous mutations and non-synonymous mutations. Sequences were recoded as follows: if compared to the reference sequence, non-synonymous residues are noted A, synonymous residues are noted S, identical residues are noted 1, non-identified residues are noted N; identical or synonymous residues are noted W.

Preferential association of two alleles at two different loci (designated pos_i_ and pos_j_) is known as linkage disequilibrium. A linkage disequilibrium coefficient *D*’ is computed with the encoded sequence dataset as explained by the group of Lewontin [29,30]. Using this coefficient, we determined the background linkage disequilibrium [31,32] correlated to the number of couples sharing a common ancestor, and whose covariation was not the consequence of functional interdependencies.

To obtain *D*’, coefficient *D* is first computed:

$$D=\left(\frac{\left(1_{i}\cdot S_{j}\right)+\left(S_{i}\cdot 1_{j}\right)+\left(S_{i}\cdot S_{j}\right)+\left(1_{i}\cdot 1_{j}\right)}{N}\cdot \frac{\left(A_{i}\cdot A_{j}\right)}{N}\right)-\left(\frac{\left(A_{i}\cdot 1_{j}\right)+\left(A_{i}\cdot S_{j}\right)}{N}\cdot \frac{\left(1_{i}\cdot A_{j}\right)+\left(S_{i}\cdot A_{j}\right)}{N}\right)$$

*N* = total number of sequences in the alignment that possess an AA at pos_i_ and pos_j_; *D* is then normalized as follows:

$$\mathrm{if}\;D<0\;\mathrm{then}\;D_{max}=\min\left\{freq\left(W_{i}\right)\cdot freq\left(W_{j}\right);freq\left(A_{i}\right)\cdot freq\left(A_{j}\right)\right\}$$

$$\mathrm{if}\;D>0\;\mathrm{then}\;D_{max}=\min\left\{freq\left(W_{i}\right)\cdot freq\left(A_{j}\right);freq\left(A_{i}\right)\cdot freq\left(W_{j}\right)\right\}$$

Finally: $D^{\prime }=\frac{D}{D_{max}}$

Positions are considered interdependent when *D*’ value is superior to 1.5 or inferior to 0.5. A couple is determined as unlinked to the background noise when:

$$\frac{D\prime \left(A-A\right)}{D\prime \left(S-S\right)}>2.\;\mathrm{To}\;\mathrm{simplify}\;\frac{D\prime \left(A-A\right)}{D\prime \left(S-S\right)}\;\mathrm{is}\;\mathrm{written}\;D_{\frac{AA}{SS}}^{\prime }$$

### 2.6. Characterization of Interdependent Pairs into Compensatory Mutations (CM) or Synthetic Lethals (SL)

A pair of residues is defined as a pair of CM, when the appearance of first mutation changes the phenotype (here, an essential function of the protein) and a second mutation re-establishes the original phenotype. On the other hand, an SL pair is defined by two mutations which, when they appear separately have the wild-type phenotype and when they appear together, actually change this phenotype. Using the coefficient of dissimilarity ξ described by Petitjean et al. [11], each interdependent pair previously determined is differentiated into CM or SL. If one considers a pair of residues A at pos_i_ and B at pos_j_ (A and B can be any AA), in which the number of residue pairs theoretically calculated is higher than the number of couples observed, then this coefficient is negative and corresponds to a pair of SL. Otherwise it is positive and corresponds to a pair of CM:

$$\mathrm{if}\;N_{obs}\left(A_{i},B_{j}\right)\geq N_{ex}\left(A_{i},B_{j}\right)\;\mathrm{then}\;\xi \left(A_{i},B_{j}\right)=+\chi^{2}\left(A_{i},B_{j}\right)$$

$$\mathrm{if}\;N_{obs}\left(A_{i},B_{j}\right)<N_{ex}\left(A_{i},B_{j}\right)\;\mathrm{then}\;\xi \left(A_{i},B_{j}\right)=-\chi^{2}\left(A_{i},B_{j}\right)$$

*χ*^2^(*A_i_*, *B_j_*) is calculated as in the Noivirt study.

### 2.7. Determination of Binding Sites Generating Little Resistance to a Small Drug Molecule

Networks of spatially close (less than 10 Å) SLs and invariant positions are defined as targets. The Fpocket software [33] is used to determine pockets in the quaternary structure reference. To find small pockets we reduced minimum and maximum radii or alpha spheres to 2.5 Å and 4 Å respectively. Pockets with a volume between 60 Å^3^ and 500 Å^3^ and at least five residues of one of the targets (groups of “SL + Invariants”) [28] were retained.

### 2.8. Accession Numbers

The protein database accession number of the H1N1 HA is 1RU7 and 3HMG for H3N2. The protein and nucleotide sequences of the HA were downloaded from the flu.org database site.

## 3. Results

At its surface, influenza A (H1N1 and H3N2) expresses a crucial protein for viral entry into its host cell [15,16,17], the antigenic HA glycoprotein that binds to sialic acid on the host cell membrane.

### 3.1. Prediction of Therapeutic Targets In Silico

To define protein regions as potential targets for drugs and avoiding therapeutic escape, we sought to highlight sets of spatially close SL couples and invariant positions on the HA surface.

For this, we chose to follow a remodeled version of the seven step protocol previously described by Petitjean et al. [11]. Indeed the initial procedure depicted four tests performed in parallel, three statistical nonparametric evaluations (Fisher, *D*’ and *r*^2^), and the semiparametric *χ*^2^ test. The three nonparametric analyses were implemented on the same protein alignment as the *χ*^2^ test but this alignment was recoded into a simpler form (only two states) for each position: mutated or not mutated compared to the reference sequence. Consequently these three non-parametric evaluations provide redundant and less descriptive information. We therefore decided to only perform the *χ*^2^ test on an AA alignment containing the qualitative and quantitative data.

The steps of the workflow are presented in Figure 1: first, variant positions were identified (those having more than 0.3% variability in the alignment). These variant positions were then tested as pairs using the statistical tests described in the Material and Methods section: couples responding positively to the *χ*^2^ test were considered as interdependent pairs. Using a BLD test, we discarded couples that phylogenetically derive from a common ancestor and therefore do not emanate from functional interdependencies.

Among these interdependent couples (that can be SL or CM), a dissimilarity test allowed us to identify SL couples and exclude CM pairs. Next, only couples at the surface of the protein reachable by a drug were retained.

Subsequently, only spatially close SL couples (whose residues are separated by less than 10 Å) and invariant positions (those having less than 0.3% variability in the alignment) adjacent to these couples and at the surface of the protein were retained. Finally, employing the Fpocket software [33] and the HA 3D structure, we determined the “druggability” of the retained sets of residues, so as to list the most relevant binding sites on this protein.

### 3.2. The H1N1 Case

Following this protocol, a 10,781 HA sequence alignment was built, constituted of 483 AA positions. Among them, 88 variant positions are located on the surface of the protein (162 are variants and 176 are determined with the ASA software [26] on the surface of the protein) and constitute 3828 (=88 × (88 − 1)/2) couples, while only 163 of them are on adjacent positions. Of these 163 couples, 137 are defined as interdependent when applying the *χ*^2^ test (see Materials and Methods Section 2.4.) and 59 are functionally coupled (employing the BLD test; see Materials and Methods Section 2.5.) and thereby exclude the probability of sharing a common ancestor. Forty-two couples responded positively to both the *χ*^2^ and BLD tests and hence are interdependent, spatially close and on the surface of HA.

The 42 pairs were analyzed for the quality of their interdependence. For a couple of positions, 190 (=(20 × 19)/2) possible pairs of residues could be retained, since each position can bear, at least theoretically, 20 different AAs. Thus, among these 190 positions, some pairs of residues can be SL others CM and therefore a couple of positions can be either SL or CM or both. Adopting a method defining all pairs of residues found in each pair of covariant positions as well as their quality, 25 couples of positions possess SL pairs (*χ*^2^ values are available in Table 1).

**Figure 3:**
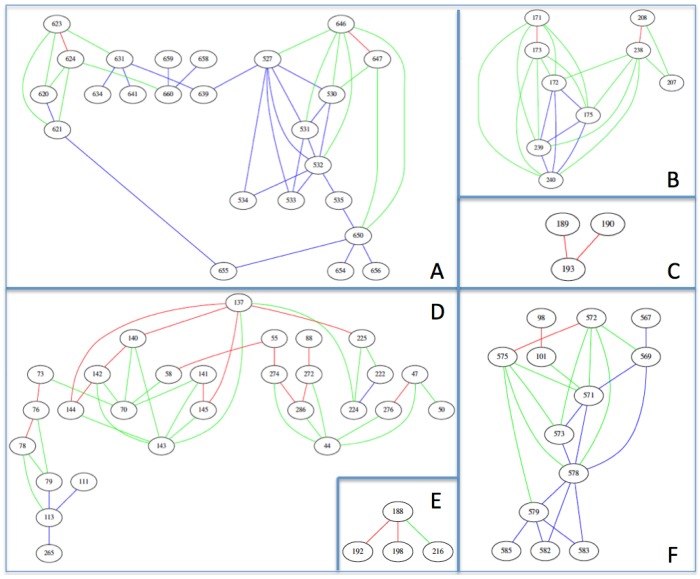
Graph representation of interactions between spatially close SL and invariant positions on the surface of influenza A H1N1 HA. Nodes are positions of HA. Edges between two positions means that these positions are spatially close (less than 10 Å) and at the surface of HA. Couples of SLs are linked by red edges. Couples with an SL and an invariant position are linked by green edges. Couples of invariant positions are linked by blue edges. Note that while the protein has only 483 positions (323 AA for HA1 and 160 for HA2), the numbering is up to 660 positions. The numbering of the residues is based on the numbering of the PDB structure of 1RU7 where the numbering is as follows, from 5 to 327 (HA1) then from 501 to 660 (HA2). Six sets of interactions are divided into six sub-graphs consisting of 24 (**A**), 9 (**B**) 3 (**C**), 28 (**D**), 4 (**E**) and 13 (**F**) positions.

To delineate our future potential targets, we must seek invariant positions in the vicinity of these 25 SLs. Figure 3 shows an undirected disconnected graph consisting of six sub-graphs. The 45 invariant positions spatially close to SLs were added on the graph. These six sub-graphs draft six potential targets. Target A composed of 24 residues: two couples of SL (623-624 and 646-647) and 20 invariant positions (527, 530, 531, 532, 533, 534, 535, 620, 621, 631, 634, 639, 641, 650, 654, 655, 656, 658, 659, 660). Target B is formed of nine residues: two couples of SL (171-173 and 208-238) and five invariant positions (172, 175, 207, 239, 240). Target C is the aggregation of two SL couples (189-190, 190-193). Target D is composed of 28 residues: four groups of SLs (73-76-78, 137-140-141-142-144-145-225, 55-58-88-272-274-286 and 47-276) and 10 invariant positions (44, 50, 70, 79, 111, 113, 143, 222, 224, 265). An SL group (188-192-198) and the invariant position 216, compose the four residues of target E. Finally, the sixth target F combines 13 residues: two couples of SLs (98-101 and 572-575) and nine invariant positions (567, 569, 571, 573, 578, 579, 582, 583, 585).

**Figure 4:**
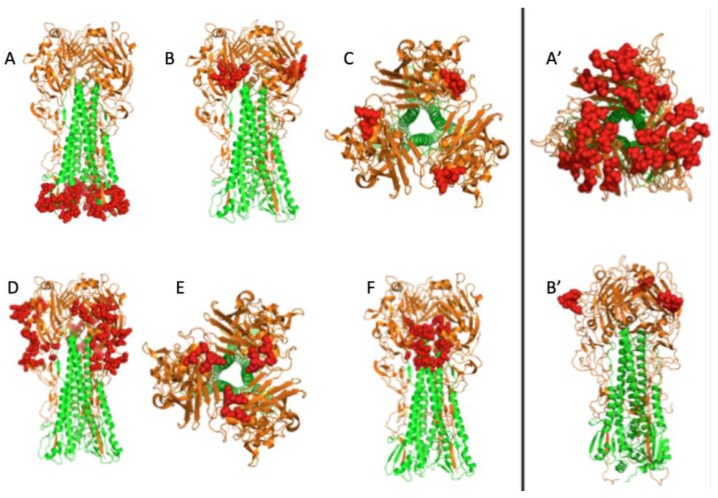
3D view of the six potential therapeutic targets on H1N1 and two on H3N2. Red spheres are the targets. HA1 subunits mainly forming the globular head of HA are in orange. HA2 subunits that mainly form the stem of HA are in green. Lists of positions of these six targets are described in Figure 3. Target A is localized at the bottom of the stem on HA2. Targets B, C, D, and E are localized on HA1. Target F is at the junction between HA1 and HA2. Target A’ and B’ are on H3N2 HA1.

Figure 4 depicts the 3D localization of these six potential targets defined by analyzing the PDB 1RU7 structure of the HA by running the PyMOL 1.8.2.0 software. Targets B, C, D, and E are located on the globular head of HA. This head is at the distal end of the protein which is at the surface of the virus and hence accessible to a potential small drug molecule. Target A is located at the bottom of the stem of HA. This area is directly in contact with the virus envelope and is certainly difficult to access. This target is mainly composed of invariant positions that can be explained by its localization. In fact, being buried implies that this part of the protein is less subject to environmental selection pressures. Target F is at the junction between HA1 and HA2. This target could be docked to block the formation of the protein quaternary structure prior to forming virions.

#### 3.2.1. Can These Pockets Tie Up Small Drug Molecules?

The six potential targets contain “SL + Invariant” positions predicted to be good spots to avoid resistance. To be of therapeutic interest, these targets should constitute binding sites, meaning pocket-shaped locations and composed of atoms that a drug-like molecule (small molecule) can bind. From the PDB three-dimensional HA structure 1RU7, Fpocket software determines protein regions that can form binding pockets. To select less escape-prone targets, pockets composed of at least five residues positioned in one of the targets previously described, were retained. As our aim was to determine pockets binding small molecules, we also restricted pocket volumes to a range between 60 Å^3^ and 500 Å^3^ and set the Fpocket parameters to a minimum radius of alpha spheres at 2.5 Å and maximum radius of alpha spheres at 4 Å. With these parameters and our criteria, five binding candidate pockets were retrieved (Table 2).

The volume site of the first pocket, containing positions 44, 47, 55, 274, and 286 is 488 Å^3^. The volume site of the second pocket is 175 Å^3^, and this pocket is composed of positions 621, 624, 655, 656, and 658. The third site is constituted by positions 624, 656, 658, 659, and 660: its site volume is 166 Å^3^. The volume of the fourth site formed of positions 571, 572, 575, 578, and 579 is 98 Å^3^. The last site containing positions 571, 572, 575, 578, 579, and 582 has a volume of 221 Å^3^.

#### 3.2.2. The Role of Hemagglutinin Domains

To be efficient therapeutic targets, two specific goals should be fulfilled, first, to adequately bind to a small molecule, and secondly, once docked with this molecule, to be able to block an important HA function. Our method attempts to achieve these two goals, the first using Fpocket, the second using SL couples and invariant positions to define these targets. To confirm the legitimacy of our method we analyzed the protein structure in detail.

**Figure 5:**
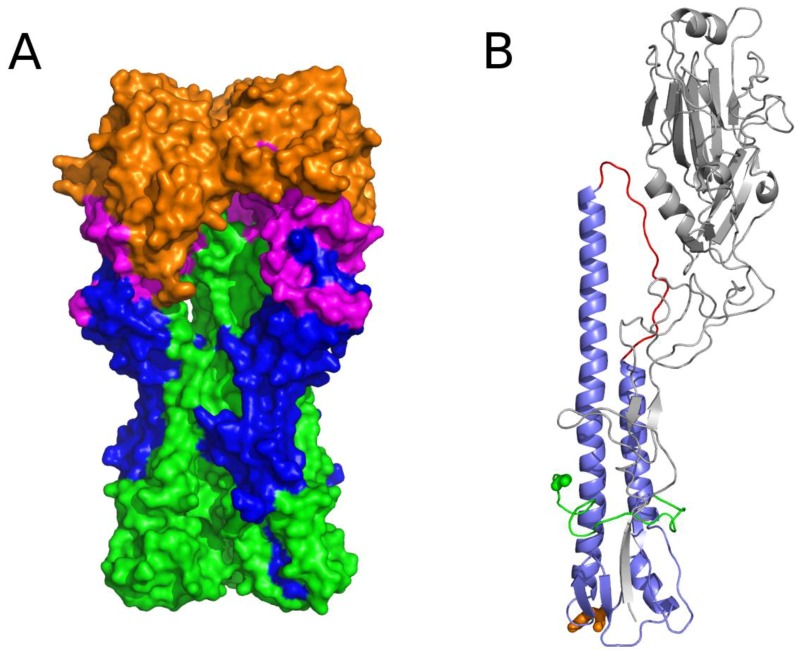
3D view of influenza virus HA protein features. (**A**) 3D representation of the 1RU7 protein. Each monomer has a globular head (located on HA1) at the surface of the virus envelope and a stem (located on HA2) bound to the virus envelope. HA1 carries the receptor binding domain in orange, the vestigial esterase domain in magenta, and the F’ subdomain of the fusion domain in blue. HA2 is constituted of an F subdomain of the fusion domain in green; (**B**) 3D representation of a monomer of the HA protein 1RU7. The globular head HA1 is in gray. The N-terminal end of HA2 is in green and the C-terminal end is in orange. The fusion peptide which binds to the host cell membrane is part of the N-terminal end and is in green. The B-loop is in red. Other colors are features of the HA2 stem.

The wild-type HA homotrimer is coded by the fourth RNA segment of the influenza virus. Each monomer is composed of two polypeptides, originating from the cleavage of a single HA polypeptide of 549 AAs that eliminates one AA. The first one, HA1 (corresponding to the former 327 AAs at the N-terminal end of the HA uncleaved polypeptide) forms a globular head, and the second one, HA2 (corresponding to the former 222 AAs at the C-terminal end of the HA uncleaved polypeptide) constitutes the stem of the protein edifice with the N-terminal part of HA1 (for the global HA 3D structure, see Figure 5). From the head to the base of the stem, three different regions compose HA. The head and the intermediate part are solely composed of AAs of HA1. The head constitutes the receptor binding (RB) domain that encloses the sialic acid binding sites. The B, C, and E targets are localized in this RB domain. The intermediate part of the protein, located between the RB domain and the foot of the stem consists of a vestigial esterase domain, and is the location of the D target. The fusion domain is in the stem and is constituted of F (on the HA2 polypeptide) and F’ (on the HA1 polypeptide) subdomains (Figure 5) [16]. Finally, the A target is localized at the bottom of the stem.

The main role of HA is to allow entry of the influenza virus into its host cell by endocytosis. To achieve this, the protein can fuse the viral envelope to the endosomal membrane. During this mechanism, the head of the HA binds to specific sialic acid host cell membrane proteins. The polypeptide HA2 then adopts multiple extremely different conformations from its pre-fusion conformation. One of the significant changes in the secondary structure of HA2 allows binding of the fusion peptide to the endosomal membrane.

**Figure 6:**
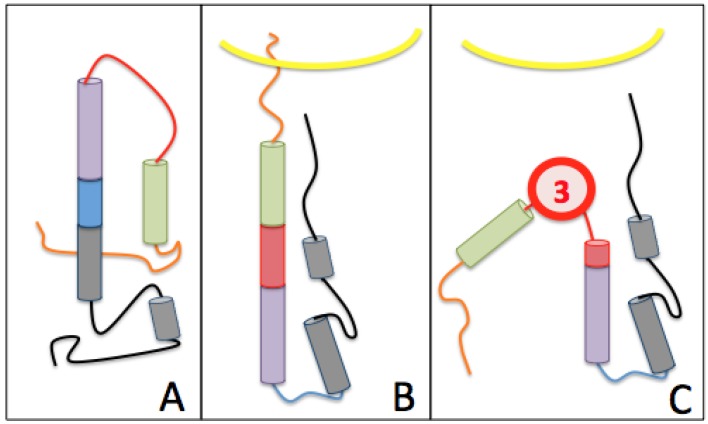
Proposed model of fusion disruption by a small molecule docked on target 3. Only the HA2 polypeptide is shown for clarity. It consists of 6 distinct parts: the fusion peptide located at the extremity of the polypeptide N-terminus is orange, followed by zone A in the helix (in green), loop B is in red, zone C in the helix is in purple, the kink in blue and zone D in black at the C-terminal end of the protein. (**A**) HA2 is in its pre-fusion conformation; zone B is in the form of a loop, and the kink area as a helix; (**B**) The HA2 peptide is in its post-fusion conformation where area B is in the form of a helix, and the kink region in the form of a loop; (**C**) The small molecule, a future potential drug, specified by a red circle noted 3, binds to a portion of loop B. Loop B therefore cannot form an entire helix. The amino acid sequences of these different zones are defined in Figure 2.

This is due to the “spring-loaded” mechanism of the HA2 B-loop area, which is a loop-to-helix transition [34,35,36] (Figure 6A,B). Once the virus reaches a low pH environment such as the endosome pH, the hydrophobic fusion peptide located at the N-terminal end of HA2 is released [15,16,17,37] (in green in Figure 5B), which is essential for HA membrane fusion activity.

#### 3.2.3. A Function for the Described Pockets?

**Figure 7:**
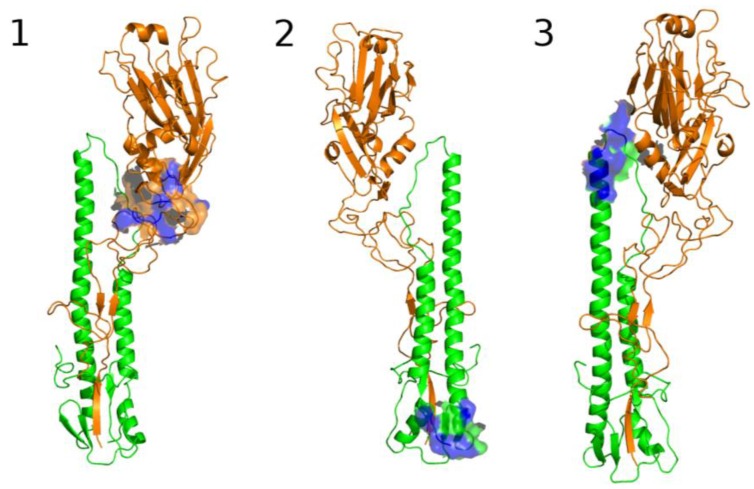
3D view of the 3 potential therapeutic targets for drugs. Targets are represented as surfaces, they are shown on one monomer of HA. Polypeptide HA1 of HA is in orange and polypeptide HA2 is in green. Target residues described in Figure 3 and Figure 4 are in blue. Target 1 (Pocket #9) is located at the globular head of HA, its residues are mainly located on HA1. This target has residues of target D described in Figure 3 and Figure 4. Target 2 (Pocket #67) is located at the bottom of the HA stem in HA2 and has residues of target A described in Figure 3 and Figure 4. Target 3 (Pocket #83) is located in the fusion domain, in contact with the HA2 B-loop that plays an essential role in membrane fusion. This target has residues of target F described in Figure 3 and Figure 4.

**Figure 8:**
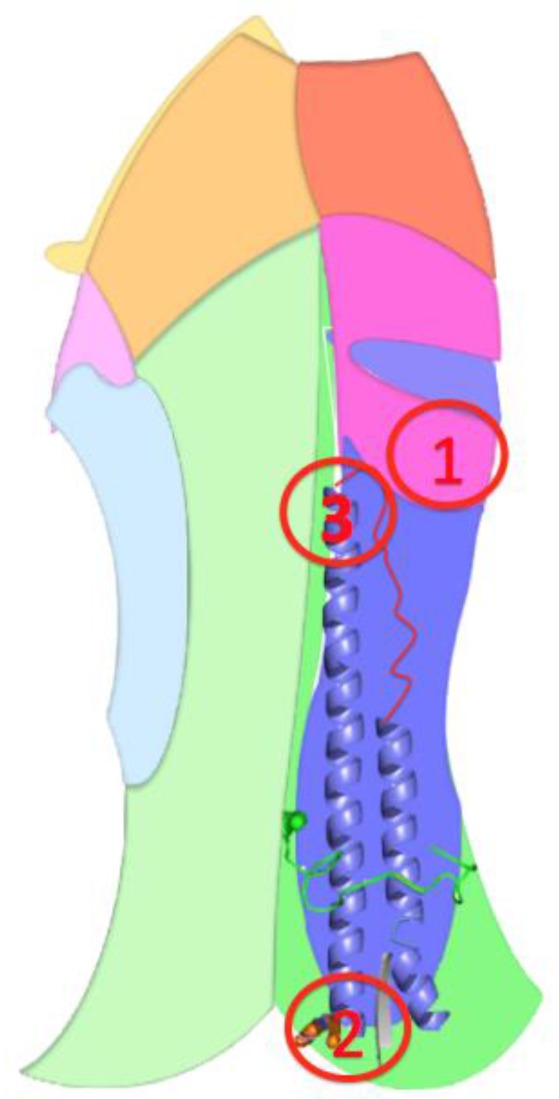
Potential targets and HA functions. This HA scheme describes the three subunits of the protein (3 different shades of the same color to differentiate subunits; if there are only two shades, this means that the third subunit is not visible in the 2D projection of the 3D structure) and its various protein domains. The receptor binding domain borne by HA1 is in orange. Vestigial esterase, the field carried by HA1 is pink. The F fusion domain borne by HA1 is in blue. The F fusion domain borne by HA2 is in green. The subdomains F’ and F form the fusion domain. Some important parts to understand the fusion process are also noted: the green loop is the fusion peptide located at the N-terminus of HA2, the residue at the C-terminus of HA2 is orange and the beta-sheet at the N-terminus of HA1 is gray. The B-loop is red. The alpha helices of HA2 are blue. The three potential targets are designated 1, 2, and 3 in red circles and are depicted only on one subunit for clarity.

The five Fpocket binding site candidates can be located on different HA domains. Pocket #9 is mainly located on HA1 in the vestigial esterase domain. Pocket #32 and #67 are located in the fusion domain, at the bottom of the protein stem, they have the same number of SLs and invariant positions (Table 2) and also have a comparable pocket volume. Pocket #45 and #83 are located in the same area as the fusion domain in the intermediate part of the HA protein. The only difference between these two pockets is that pocket #83 has an extra “SL” in comparison with pocket #45. Therefore, the analysis is refined with respect to three candidate binding sites, pocket #9 renamed the first pocket, pocket #67 renamed the second pocket and pocket #83 renamed the third pocket (respectively Figure 7 and Figure 8).

##### The Third Pocket Could Block the Spring-Loaded Transition

The third pocket (previously pocket #83, positions highlighted in yellow in Figure 2) is located in the upper part of the fusion domain and is in contact with the B-loop of HA2 (Figure 7 and Figure 8). During the membrane fusion process, after the dissociation of the HA1 monomers, the HA2 polypeptides undergo several different conformations in order to bring the two membranes closer to each other. The “spring-loaded” transition, an unavoidable step, allows the loop B to adopt a helical secondary structure, and the kink region to form a loop rather than the initial helix (Figure 6A,B). As such, this potentially important target could prevent the membrane fusion mechanism. Therefore we propose a model in Figure 6C, where docking a small drug molecule in this target can possibly block the “spring-loaded” mechanism thereby preventing the conformational change of the B-loop and hence blocking the essential function of HA which is membrane fusion.

##### The First Pocket Could Block Structural Changes

As already indicated above, the first pocket (previously pocket #9, positions highlighted in light blue in Figure 2) is mainly located on HA1 in the vestigial esterase domain (Figure 7 and Figure 8). At the beginning of the membrane fusion process, HA unfolds its globular head meaning that the three monomers of HA1 dissociate from one another. While it was thought that this dissociation was performed without structural changes [15], studies of pre-fusion mechanisms have shown that the interaction between HA1 and HA2 could pass through an early intermediate stage having a different 3D structure and allowing the release of loop B [36].

The conformation of this intermediate at the pH of fusion shows that the vestigial esterase domain would acquire a new 3D conformation acting as a relatively flexible linker between the rigid receptor binding domain and the F’ fusion subdomain. Thus, the docking of a small molecule in this pocket located at the surface of the vestigial esterase domain, could sterically prevent the receptor binding domain from approaching the F’ subdomain thereby avoiding the fusion mechanism. This hypothesis is supported by a study that shows that membrane fusion depends on a conformational change. Indeed a double mutant allowing the formation of a Cys-Cys bridge thereby blocking the possibility of conformational changes of the globular head possesses an extremely diminished [38] capacity to fuse.

##### The Second Pocket Could Block the Fusion Mechanism

The second pocket (previously pocket #67, positions highlighted in grey in Figure 2) is located at the bottom of the fusion domain composed of the C-terminal end of HA2 and the N-terminal end of HA1 (Figure 7 and Figure 8). Membrane fusion is accomplished by bringing together the viral envelope and the endosomal membrane of the host cell. The role of the fusion peptide is to be inserted into the endosomal membrane, and the C-terminal end of HA2 has a transmembrane peptide buried within the viral envelope [15,16,17,37]. Therefore the two terminal ends of HA2 are important regions in the fusion process. This pocket is in the vicinity of this C-terminal end and could be an important spot to block the fusion mechanism, but as indicated in the beginning of this section, the HA 3D structure used in this study does not possess the 62 AAs at the C-terminal end of HA2, making it impossible to fully investigate this region to identify new targets. The pocket is located at the surface of the protein but the C-terminal end is buried in the viral envelope that consequently might be difficult to access by a small molecule.

### 3.3. Comparison of H1N1 and H3N2 Strains

H1N1 and H3N2 do not seem to share the same evolutionary history. Indeed H1N1 is responsible for the 1918 epidemic, and has reappeared regularly since then in human species (except for the 1920s and the 1960s) while studies of anthropology show that H3N2 would have been found in humans in the 1890s [39], and no longer seems to have infected the human species until the 1960s, but rather ducks and horses [40] as well as birds and swine [41,42]. Thus the phylogenetic history of these two strains is very different, compromising the possibility of carrying out a single study where all the H1N1 and H3N2 sequences would constitute a same alignment. We therefore decided to conduct a new study dedicated to H3N2 using the same workflow as for H1N1 but from an HA H3N2 alignment of 12,225 sequences. A comparative study of the 3HMG (H3N2) and 1RU7 (H1N1) primary reference sequences show very low percentages of identity and homology (43% and 61% respectively), an observation that supports the hypothesis of a different evolution of the two strains. Our results support this same postulate since from the H3N2 alignment we only detect 37 pairs of covariant positions whereas H1N1 has 88 pairs. Yet their quaternary structure is very similar and their function is identical; this would rather suggest an evolutionary process that may be different with respect to their primary sequence but similar in terms of quaternary structure and function. The question is whether these two strains followed the same evolutionary pathway where H3N2 would be less advanced or whether the evolution of these two viruses differed very early after the first specimen of the species appeared, leaving no possibility of following the same mutational path, but maintaining a selection pressure on the function of the molecule. After applying to our dataset the *χ*², BLD and dissimilarity tests, we were able to describe two sub-graphs which draft two potential targets (Figure 4A’,B’). Target A’ is composed of 23 residues (124, 126, 128, 129, 131, 132, 133, 156, 157, 158, 159, 160, 163, 165, 167, 187, 188, 189, 192, 193, 197, 198, and 199) and Target B’ is formed of eight residues (137, 140, 141, 142, 143, 144, 145, and 224). The detection of these two targets corroborates our initial results, which predicted that SL groups would be limited in number because there was little covariance in general. The first target H3N2 (Figure 4A’) is in fact the conjunction of two H1N1 targets (Figure 4C,E) and the second target is included in the proposed D target for H1N1 (Figure 4B’). These results bear the thesis of a shared evolution in terms of function despite the primary sequence of these two proteins.

### 3.4. Which Ligand Could Bind This Third Target?

The spring-loaded process is an essential step in viral infection. Indeed, it has been shown that a membrane fusion inhibitor links this region to inhibit this process [24,25]. Another study revealed that two different small molecules inhibiting the entry of the virus into the eukaryotic cell can prevent a monoclonal antibody from binding to this region (position: vertically hashed in Figure 2) [25]. These three different targets are fixed in the same region, and mainly in zone A, which is on the N-terminal side of loop B. The third target described here is on the C-terminal side of loop B. The simplest hypothesis to explain their mechanism of action would be that the attachment of a small molecule could prevent the structural transition from loop to helix of zone B.

**Figure 9:**
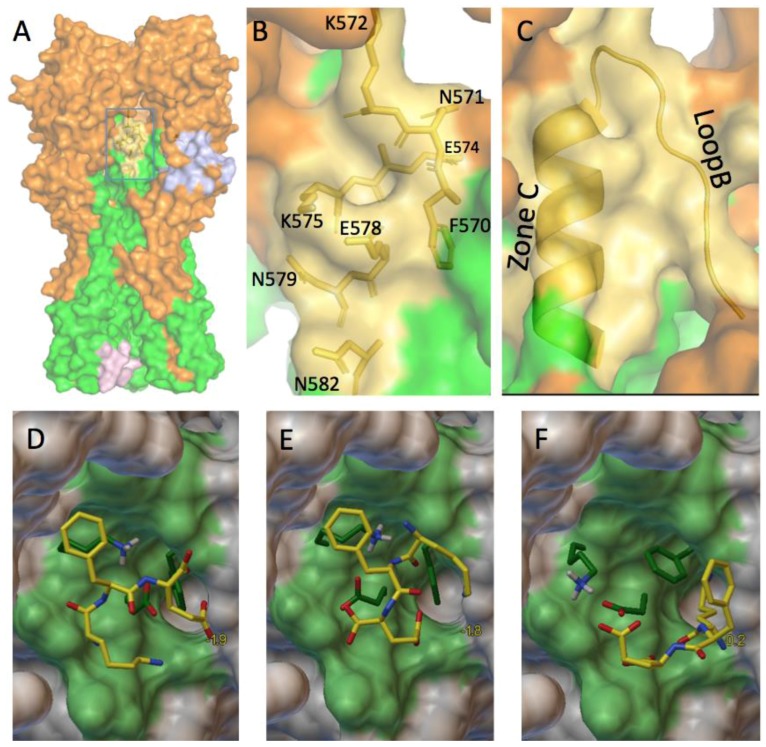
A possible future tripeptide drug. (**A**) A 3D view of H1N1 hemagglutinin. In yellow, the third target; (**B**) An enlarged view of the blue box of Figure 9A depicting the amino acids constituting the third target; (**C**) An enlarged view of the blue box of Figure 9A where the helix C and the loop B are positioned; (**D**–**F**) Three proposed docking of the tripeptide KFE on the third target.

Panel 9C (enlarging the frame of Figure 9A) describes the three-dimensional structure of the region prior to virus binding to the host cell: zone C is helically structured and zone B is looped. To overcome the spring-loaded process, two structural changes must be stopped: zone B must not adopt a helical structure and the two regions B and C must not line up. To try to prevent this from occurring, we wish to stabilize this region in its helix-C and loop-B form. Figure 9B (enlarging the frame of Figure 9A) shows the position and type of AAs that make up this target. These AAs (with the exception of F570) are polar, and four of them (two lysines and two glutamic acids) are charged (at a physiological pH of 7.4). We propose to build a tripeptide, KFE, consisting of two charged AAs (at a physiological pH) which can bind to the polar AAs of the target and a rather lipophilic AA therefore apolar which could have affinity for long carbon chains or benzene ring. This tripeptide could therefore bind F570 AAs of loop B and K575 and E578 of zone C to maintain these two secondary structures in a position that does not allow the virus to fuse to the cell membrane. The choice of a peptide rather than another small molecule is important to ensure the non-toxicity of the possible future drug. We first used the Avogadro software to find the most stable conformer of the KFE tripeptide using the universal force field. The energy of this conformer is 300 kJ/mol. We generated a new conformer of equivalent energy whose structure is closer to that required to bind AAs F570, K575 and E578 as they are positioned in the 1RU7 structure. Using the Autodock suite (Autodock tool and Autodock vina), we performed docking experiments between this conformer and the 1RU7 protein with the following parameters: the tripeptide can adopt a flexible structure as well as the three AAs F570, K575, and E578, the rest of the protein bearing a rigid structure. Three different docking are obtained, represented in Figure 9. DEF whose free energy ΔG_0_ are −1.9, −1.8, and 0.2 kcal/mol, respectively.

## 4. Discussion

In this study we describe an in silico method to find therapeutic targets, that once bound by a drug, would leave the virus fewer opportunities of escaping. The first step was to discover the pairs of residues that are interdependent, performing statistical tests. It is interesting to discuss the discriminatory nature of these tests. Indeed *χ*^2^ is not as selective as BLD with 84% of positive answers to the test and only 36% for BLD. The 163 pairs of positions were chosen because they are at the surface of the protein and thus are certainly more exposed to external selection pressures. We can therefore hypothesize that the choice of studying the exposed positions already provides a first selection of most variant positions that are not buried within the protein. Hence, the results of the BLD test show the importance of discriminating interdependencies due to the existence of a common ancestor of covariance for functional reasons.

Six potential therapeutic targets are described in this manuscript, three of which correspond to real pockets, and could be future locations for the attachment of small drug molecules. Two of these pockets are particularly interesting as they are in contact with an extremely important region to allow membrane fusion. We present a model (Figure 6) showing that the third pocket described here could prevent the secondary structure change of the HA2 zone B. Indeed, this area forms a loop before fusion of the virus with the host cell and must adopt a helical structure to allow the attachment of the peptide to the endosomal membrane. Once linked to a small molecule, this target could durably prevent the essential membrane fusion function of the virus and therefore make it non-pathogenic. This is why we have chosen to design a tripeptide (KFE) whose physico-chemical properties should make it possible to bind this target on residues E578, F570 on zone C, and K575 on loop B. These preliminary molecular modeling results describe three possible protein-ligand bindings. The first two proposed dockings possess free energy showing a non-negligible affinity between the small molecule and the protein (the free energy of the third is too large to show a real affinity between these two molecules). These energies correspond to dissociation constants between 10^−2^ M and 10^−3^ M, which is of course too large to analyze these results as final but sufficiently weak to engage in a more advanced chemo-informatic study. Firstly, molecular dynamics experiments would allow a description of the conformation of 1RU7 having the lowest energy and therefore the most stable conformation. This new conformation could be used in new drug design experiments leaving the whole protein flexible, which would possess more degrees of freedom to bind the tripeptide KFE.

The vestigial esterase domain seems to act as a hinge between the receptor binding domain and the F’ subdomain. Once bound to a small molecule, the first pocket located in this area could prevent the conformational change allowing the receptor binding domain to come closer to the F’ subdomain.

The comparative study of H1N1 with H3N2 allowed us to show that these two subgroups evolved in a similar way in terms of quaternary structure and function, since the two targets detected during the examination of H3N2 are part of the six targets discovered for H1N1. However, the fact that the number of couples of covariants for H3N2 is 2.4 times lower than for H1N1, and that the number of targets found is also lower for H3N2, imply that H3N2 is less advanced in this common evolutionary path and shows (if still necessary) that the function is not carried solely by the primary sequence of the proteins.

We also consolidated the statistical test required for the detection of therapeutic targets, making this method simpler and thus easier to use by other researchers.

To confirm the existence of these targets by biological tests, a laboratory specialized in influenza could mutate the invariant groups and calculate the residual viral fitness. Indeed, invariance groups are defined as essential for viral function. Thus, the mutant replication rate should be much reduced.

Finally, if viral fitness experiments in biology and chemo-informatic studies are successful, inactivation of A H1N1 influenza virus with this small molecule could be considered.

Many resistance mutations appear on the M2 transmembrane protein [43] that forms an ion channel to change the internal pH of the virus. The genetic material of the virus can be released into the target cell only when HA adopts a conformation that cannot be achieved when the internal pH of the virus is not more acidic. This suggests that it would surely be interesting in the future to seek SLs on the M2 protein.

There are mutations in HA causing resistance to treatment aimed at the NA protein [35]. Currently the anti-flu treatments used in the West are targeted mainly toward NA [44]. Hence, it would be interesting to investigate possible intergenic SLs between NA and HA.

It is the first time this method has been used to search for therapeutic targets on viral envelope surface proteins. Indeed, the great variability of these proteins makes it difficult to align their sequences. The alignment presented here can be exploited to initiate the development of a new SL search method to identify novel peptide vaccines. Indeed, it is commonly accepted that vaccines developed against influenza are efficient only during one season. This method could therefore allow the development of efficient vaccines over the long term.

In conclusion, these results present an elementary method that can be widely developed, in bioinformatics on other proteins or other viruses, in chemo-informatics to design corresponding drugs, in biology for fitness tests and finally can be used for purposes of vaccinology and have allowed us to build a model possibly blocking the viral fusion mechanism.

## Figures and Tables

**Table 1:** H1N1 hemagglutinin (HA) pairs of synthetic lethals (SL) and compensatory mutations (CM).

AAi; AAj		*N_obs_*	*N_ex_*	ξ		AAi; AAj		*N_obs_*	*N_ex_*	ξ
*i* = 47; *j* = 276	KD	49	133.66	−53.63		*i* = 189; *j* = 193	ES	0	106.39	−106.39
*i* = 55; *j* = 58	HN	9	99.86	−82.67			AA	0	83.22	−83.22
*i* = 55; *j* = 274	HQ	4	5.87	−0.60			GS	2	82.96	−79.01
	HP	10,722	10,507.82	4.37			RS	0	5.86	−5.86
*i* = 73; *j* = 76	PT	20	26.41	−1.56			SS	3	5.86	−1.39
*i* = 76; *j* = 78	TK	0	100.76	−100.76			AN	17	22.00	−1.14
	TE	2	74.34	−70.40			AS	10,535	10,323.53	4.33
	PS	3	15.60	−10.17		*i* = 190; *j* = 193	NS	3	7.81	−2.96
	TS	10,757	10,545.06	4.26		*i* = 208; *j* = 238	RK	32	40.81	−1.90
*i* = 88; *j* = 272	DD	3	5.87	−1.40		*i* = 272; *j* = 286	DL	0	6.84	−6.84
	SD	10,505	10,290.91	4.45			EK	3	5.66	−1.25
*i* = 98; *j* = 101	YN	0	42.02	−42.02			DE	1810	1771.36	0.84
	NN	5	13.93	−5.72			DK	8927	8744.39	3.81
*i* = 137; *j* = 140	VP	3	11.67	−6.44		*i* = 274; *j* = 286	PL	0	6.79	−6.79
*i* = 137; *j* = 144	VA	6	11.67	−2.75			TK	20	16.18	0.90
*i* = 137; *j* = 145	VK	3	11.70	−6.47			PE	1805	1758.74	1.22
*i* = 137; *j* = 225	TE	0	5.61	−5.61			PK	8852	8682.10	3.32
*i* = 140; *j* = 142	PK	0	95.33	−95.33		*i* = 572; *j* = 574	HR	9	94.94	−77.79
	PN	0	77.82	−77.82		*i* = 623; *j* = 624	RT	5	22.44	−13.56
	PS	3	5.84	−1.38		*i* = 646; *j* = 647	DT	32	36.77	−0.62
*i* = 141; *j* = 145	YK	11	29.25	−11.38						
*i* = 142; *j* = 144	KA	0	95.28	−95.28						
	NA	0	77.78	−77.78						
*i* = 171; *j* = 173	DE	17	198.23	−165.68						
*i* = 188; *j* = 192	SR	0	71.14	−71.14						
	SK	0	68.38	−68.38						
	TR	0	29.32	−29.32						
	TK	0	28.18	−28.18						

*i* and *j* are positions of covariant positions of influenza A H1N1 HA; AAi and AAj: amino acids at positions *i* and *j*; *N_obs_*: number of sequences where AAiAAj is observed; *N_ex_*: theoretical number of sequences where AAi and AAj should be observed if they are not correlated; ξ: dissimilarity coefficient computed for the AAiAAj pairs (formula in Materials and Methods); SL: synthetic lethal. CM: compensatory mutation.

**Table 2:** Binding site candidates of H1N1 HA.

Pockets	SL + Inv	SL	Inv	Targets	Volume (Å^3^)
#9	47, 274, 286, 44, 55	4	1	D	488
#32	621, 656, 655, 658, 624	1	4	A	175
#45	575, 571, 578, 572, 579	2	3	F	98
#67	659, 658, 660, 624, 656	1	4	A	166
#83	572, 571, 578, 575, 582, 579	2	4	F	221

Inv: Invariants.
